# Fine platinum nanoparticles supported on a porous ceramic membrane as efficient catalysts for the removal of benzene

**DOI:** 10.1038/s41598-017-16833-0

**Published:** 2017-11-29

**Authors:** Hui Liu, Chengyin Li, Xiaoyong Ren, Kaiqi Liu, Jun Yang

**Affiliations:** 1 State Key Laboratory of Multiphase Complex Systems, Institute of Process Engineering, Chinese Academy of Sciences, Beijing, 100190 China; 2 Center for Mesoscience, Institute of Process Engineering, Chinese Academy of Sciences, Beijing, 100190 China; 3 University of Chinese Academy of Sciences, No. 19A Yuquan Road, Beijing, 100049 China

## Abstract

It would be desirable to remove volatile organic compounds (VOCs) while we eliminate the dusts using silicon carbide (SiC)-based porous ceramics from the hot gases. Aiming at functionalizing SiC-based porous ceramics with catalytic capability, we herein report a facile strategy to integrate high efficient catalysts into the porous SiC substrates for the VOC removal. We demonstrate an aqueous salt method for uniformly distributing fine platinum (Pt) particles on the alumina (Al_2_O_3_) layers, which are pre-coated on the SiC substrates as supports for VOC catalysts. We confirm that at a Pt mass loading as low as 0.176% and a weight hourly space velocity of 6000 mL g^−1^ h^−1^, the as-prepared Pt/SiC@Al_2_O_3_ catalysts can convert 90% benzene at a temperature of ca. 215 °C. The results suggest a promising way to design ceramics-based bi-functional materials for simultaneously eliminating dusts and harmful VOCs from various hot gases.

## Introduction

Silicon carbide (SiC)-based porous ceramics can be prepared from inexpensive raw materials, and have been widely used to clean hot gas due to their advantageous features such as good thermal shock tolerance, high anti-fouling properties, and superior abrasion resistance^1–8^. However, besides fine particulate matters, there are a lot of volatile organic compounds (VOCs), e.g. benzene, methane, ethane, propane etc. in the hot gases, particularly for those from various cooking activities^9–18^. These VOCs directly or indirectly pollute the environment, and seriously threaten the human health, e.g. headache, respiratory and skin irritation, and even cancer^19–22^. Therefore, it would be very desirable to remove VOCs while we eliminate the dusts using SiC-based porous ceramics from the hot gases. Currently, taking into account rapidness, efficiency and energy-saving, catalytic degradation is the optimal strategy for the removal of VOCs by totally oxidizing them into CO_2_ and H_2_O over certain catalysts at a considerably lower temperature.

Aiming at functionalizing SiC-based porous ceramics with catalytic capability, we herein report a facile strategy to integrate high efficient catalysts into the porous SiC substrates for the VOC removal. We will demonstrate the deposition of platinum (Pt) nanoparticles on the alumina (Al_2_O_3_) layers, which are coated on the outer surface of tubular SiC membranes with a rectangular shape as supports for the VOC catalysts. The final products are labeled as Pt/SiC@Al_2_O_3_. We choose benzene, which is commonly used in chemical synthesis, petrochemical process, and paintings, as target toxic gas for evaluating the catalytic performance of the as-prepared Pt/SiC@Al_2_O_3_ samples. As we will confirm in the main text, at a Pt mass loading of 0.176%, the as-prepared Pt/SiC@Al_2_O_3_ catalysts can convert 90% benzene at a temperature as low as 215°C with a space velocity of 6000 mL g^−1^ h^−1^. The good performance of Pt/SiC@Al_2_O_3_ in benzene decomposition suggests that our concept for simultaneous elimination of dusts and VOCs from hot gases is feasible.

## Results and Discussion

**Figure 1:**
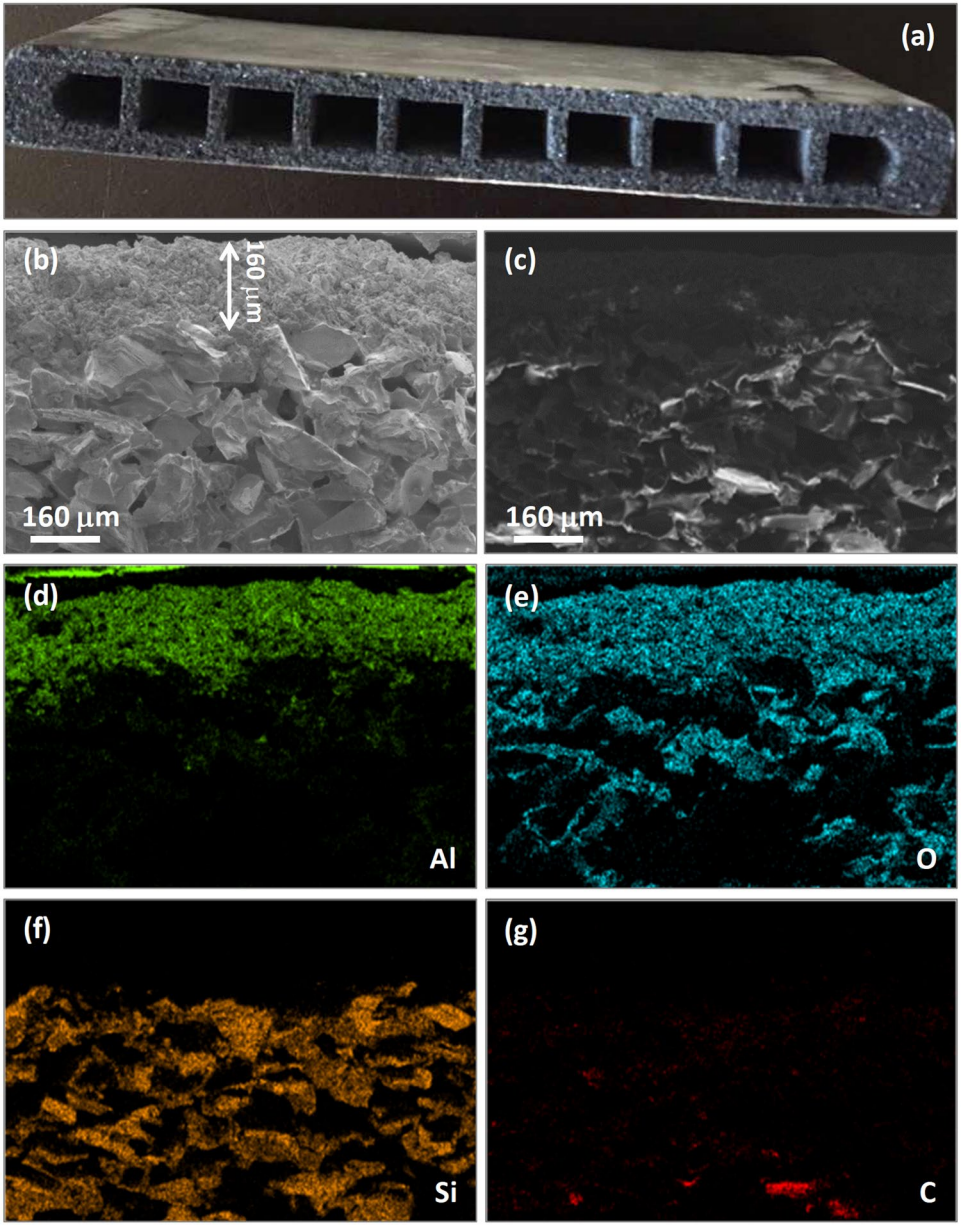
Photograph (**a**), typical SEM image (**b**), dark-field SEM image (**c**), and element mappings (**d**–**g**) of the cross-section of an as-extruded SiC@Al_2_O_3_ rectangular plate.

In this work, we aim at using a porous ceramic membrane as substrate to load an active catalyst for the removal of VOCs. Porous SiC ceramics are promising to clean fine particles from various hot gases; however, they cannot be used as substrates for VOC catalysts due to the lack of active oxygen species. Therefore, we firstly coat the SiC rectangular plates with an Al_2_O_3_ layer, which is commonly used as substrate in the catalytic degradation of VOCs^23–29^, for the catalyst loading. Figure 1a shows the cross-sectional photograph of an as-extruded SiC@Al_2_O_3_ rectangular plate, which indicates that the rough SiC surface due to larger particle sizes turns into smooth after coating with Al_2_O_3_. In addition, Figure 1a also shows that the rectangular shapes are not destroyed in the sintered process, and the SiC@Al_2_O_3_ plates are not sharp rectangular, but have truncated corners. Figure 1b shows the SEM image of the cross-section of an as-extruded SiC@Al_2_O_3_ rectangular plate, which exhibits a clear boundary between the SiC substrate and the Al_2_O_3_ layer. The thickness of the Al_2_O_3_ layer can be determined to be ca. 160 μm based on the boundary. We also obtained the dark-field SEM image (Figure 1c) of the cross-section of the same SiC@Al_2_O_3_ rectangular plate to examine the distribution of SiC and Al_2_O_3_ through elemental mapping analyses. As indicated by Figure 1d–g, the element mappings reveal that the Al and O in the rectangular plate are concentrated at one side, while the Si and C signals are distributed at the other side, suggesting the successful coating of Al_2_O_3_ on the SiC rectangular substrates.

**Figure 2:**
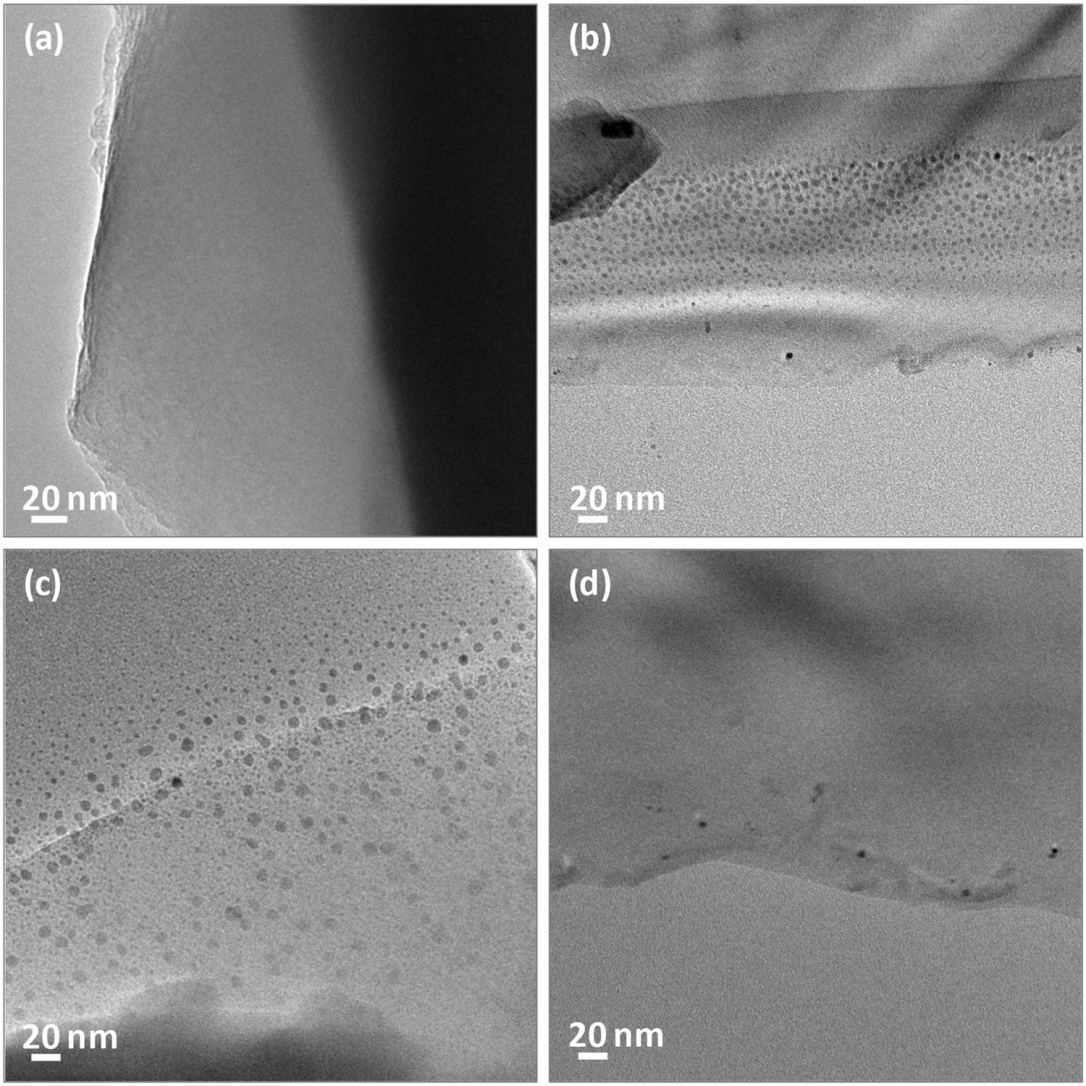
TEM images of blank SiC@Al_2_O_3_ rectangular plates (**a**), Pt/SiC@Al_2_O_3_-1 (**b**), Pt/SiC@Al_2_O_3_-2 (**c**), and Pt/SiC@Al_2_O_3_-3 (**d**).

Owing to its high activity for the oxidation of various VOCs, e.g. benzene, toluene, xylene, formaldehyde, and methane^29–36^, we chose Pt as the active component for laoding at SiC@Al_2_O_3_ rectangular plates. Figure 2 shows the typical TEM images of the blank SiC@Al_2_O_3_ rectangular plate (Figure 2a), and the specimens with Pt loading from aqueous H_2_PtCl_6_ solution (Pt/SiC@Al_2_O_3_-1, Figure 2b), from aqueous Pt colloid (Pt/SiC@Al_2_O_3_-2, Figure 2c), and organic H_2_PtCl_6_ solution (Pt/SiC@Al_2_O_3_-3, Figure 2d), respectively. Two features could be obtained from these TEM images: (1) although the aqueous/organic H_2_PtCl_6_ solution or Pt colloid is dispensed from the inner surface of the SiC@Al_2_O_3_ rectangular plates, the Pt particles are not trapped in the SiC substrates rationally because of their larger pore sizes or the intrinsic nature of SiC particles. The formed fine Pt particles with average diameter of ca. 2.58 nm (for Pt/SiC@Al_2_O_3_-1), ca. 3.32 nm (Pt/SiC@Al_2_O_3_-2), or ca. 2.24 nm (Pt/SiC@Al_2_O_3_-3) are only appeared in the Al_2_O_3_ layers; (2) In Pt/SiC@Al_2_O_3_-1, the fine Pt particles are uniformly distributed in the Al_2_O_3_ layer, while in Pt/SiC@Al_2_O_3_-2, besides the Pt particles in the Al_2_O_3_ layer, a large amount of Pt particles are also observed outside the SiC@Al_2_O_3_ rectangular plates, evincing that the pre-formed Pt particles easily fall off from the surface of Al_2_O_3_ layers. For the Pt/SiC@Al_2_O_3_-3, only few Pt particles present in the Al_2_O_3_ layers, manifesting that the strategy from organic Pt ion solution is not a good choice for loading Pt on SiC@Al_2_O_3_ rectangular plates.

We chose Pt/SiC@Al_2_O_3_-1 as a typical sample to examine the chemical state of Pt using XPS. Unfortunately, the Pt 4 f_5*/*2_ binding energy has many overlaps with that of Al 2p, preventing an unambiguous analysis of the Pt chemical state. However, the 4f 4f_7*/*2_ signal could be analyzed instead. As shown in Figure S1 of Supplementary Information (SI), the Pt 4f_7/2_ peak can be deconvoluted into two peaks with different intensities at 70.8 and 71.8 eV, respectively. The intense peak at 70.8 eV corresponds to the metallic Pt, while the weak peak at 71.8 eV may be assigned to the oxidized Pt (e.g. PtO)^37^.

The XRD patterns of blank SiC@Al_2_O_3_ rectangular plates, Pt/SiC@Al_2_O_3_-1, Pt/SiC@Al_2_O_3_-2, and Pt/SiC@Al_2_O_3_-3 are displayed in SI Figure S2. For the blank SiC@Al_2_O_3_ rectangular plates, the peaks corresponding to SiC and Al_2_O_3_ could be clearly differentiated in their XRD patterns, suggesting the presence of both SiC and Al_2_O_3_ (SI Figure S2a). However, although both TEM images and XPS analysis verify the successful loading of Pt on the SiC@Al_2_O_3_ substrates, the XRD patterns do not show the peaks corresponding to Pt phase, as seen in SI Figure S2b–d. This is probably because of tiny size of Pt particles, which significantly broaden their XRD peaks, and the low Pt content in the final specimens (≤0.5 wt% based on theoretical loading).

**Figure 3:**
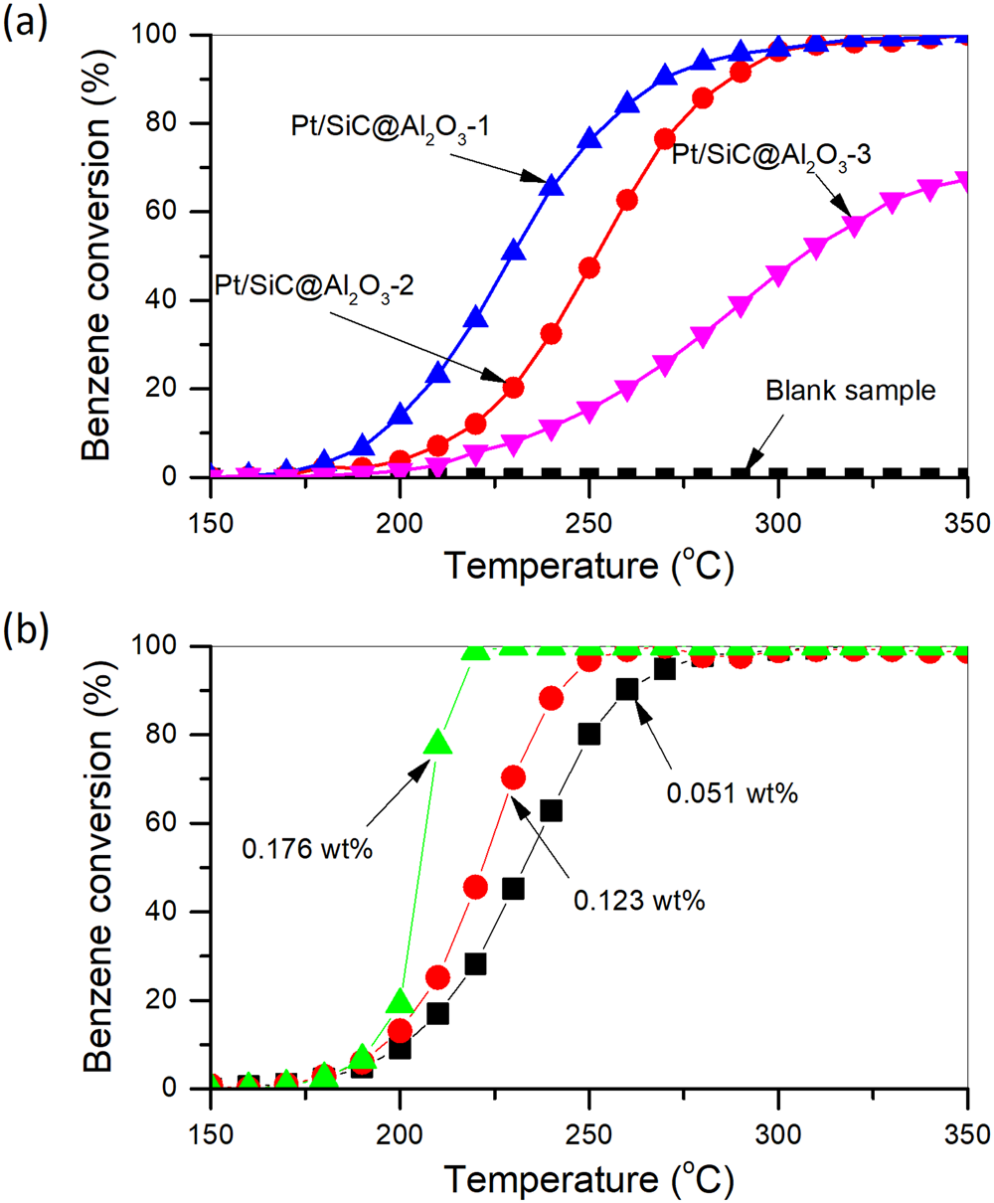
Catalytic performance of blank SiC@Al_2_O_3_ rectangular plates, Pt/SiC@Al_2_O_3_-1, Pt/SiC@Al_2_O_3_-2, and Pt/SiC@Al_2_O_3_-3 for benzene oxidation at WHSV = 60 000 mL g^−1^ h^−1^ (**a**), Effect of Pt mass loading on the catalytic activity of Pt/SiC@Al_2_O_3_ for benzene oxidation (**b**).

At a Pt mass ratio of 0.04% determined by ICP-AES, the catalytic performance of Pt/SiC@Al_2_O_3_-1, Pt/SiC@Al_2_O_3_-2, and Pt/SiC@Al_2_O_3_-3 for benzene oxidation was examined and benchmarked against blank SiC@Al_2_O_3_ rectangular plates. As illustrated by Figure 3a, the blank SiC@Al_2_O_3_ rectangular plates hardly have activity for the benzene oxidation, while all those plates with Pt loading can promote the oxidation of benzene at elevated temperature. The temperature of 10% (T_10_), 50% (T_50_), and 90% (T_90_) benzene conversion are listed in SI Table S1. As exhibited, the Pt loading strategies have apparent effect on the activity of Pt/SiC@Al_2_O_3_ specimens for benzene oxidation. The temperatures corresponding to 10%, 50%, and 90% benzene conversion for Pt/SiC@Al_2_O_3_-1 are 194.2, 229.5, and 268.2 °C, much lower than those for Pt/SiC@Al_2_O_3_-2 and Pt/SiC@Al_2_O_3_-3, indicating Pt/SiC@Al_2_O_3_-1 is most active among all catalysts for benzene oxidation. The catalytic evaluation is consistent with the TEM observation (Figure 2b), which proves that the fine Pt particles are uniformly distributed on the Al_2_O_3_ layer in Pt/SiC@Al_2_O_3_-1 specimen. It is worthy to note that in this study, we do not adopt improved strategies, such as alloying with other metals/metal oxides^38,39^, adding grapheme oxides^28^, searching more suitable substrates^40–42^, or controlling particle morphologies^43^, to enhance the catalytic properties of Pt specimen for benzene oxidation. Instead, we aim at functionalizing the SiC-based porous ceramics with catalytic capability by integrating them with Pt nanoparticles, so that the VOCs in hot gases can be simultaneously removed when we use porous SiC ceramics to eliminate the dusts. We compared the activity of Pt/SiC@Al_2_O_3_-1 with other noble metal nanoparticles supported on various metal oxide substrates for benzene oxidization. As summarized in SI Table S2, the T_90_ of Pt/SiC@Al_2_O_3_-1 specimen is comparable with those reported for other noble metal-based catalysts associated with benzene oxidation^38–42^, although low Pt loading is adopted in our studies. Analogous to the γ-Al_2_O_3_-supported dendritic Pt systems, the electronic interaction between the Pt nanoparticles and the Al_2_O_3_ layer make the oxygen more active, thus favorable for the oxidation of benzene^43^. In addition, in comparison with the much larger Pt nanodendrities (ca. 20 nm in overall size) on Al_2_O_3_ substrate^43^, the fine size (ca. 2.6 nm in diameter) and high dispersity of Pt nanoparticles in the Al_2_O_3_ layer may also have contribution to their high catalytic activity for benzene oxidation. As reported by Li *et al*.^28^, the Pt supported on Al_2_O_3_ substrates with mass ratio of 1% has lower T_90_ (ca. 150 °C–170 °C) than that of Pt/SiC@Al_2_O_3_-1. However, they added a small amount of reduced graphene oxide (rGO) to modify the Pt catalyst, and the electronic interaction between rGO and Pt might be favorable for decreasing the temperature for benzene conversion.

Encouraged by the good activity of Pt/SiC@Al_2_O_3_-1 specimen, we increased the mass ratio of Pt on the SiC@Al_2_O_3_ rectangular plates through aqueous salt method (Strategy I), and tested the catalytic performances of the as-obtained samples for benzene oxidation. As shown by Figure 3b, with the increase of Pt mass ratio from 0.051% to 0.176%, the temperature for 90% benzene conversion (T_90_) decreases by ca. 45 °C (260.1 °C, 242.5 °C, and 215.2 °C for Pt/SiC@Al_2_O_3_ with Pt mass ratio of 0.051%, 0.123%, and 0.176%, respectively). This suggests that we can maximize the catalytic performance by increasing the Pt mass loading on the SiC@Al_2_O_3_ substrates. However, the Pt loading needs to balance the cost carefully so that an optimal Pt/SiC@Al_2_O_3_ specimen could be obtained.

**Figure 4:**
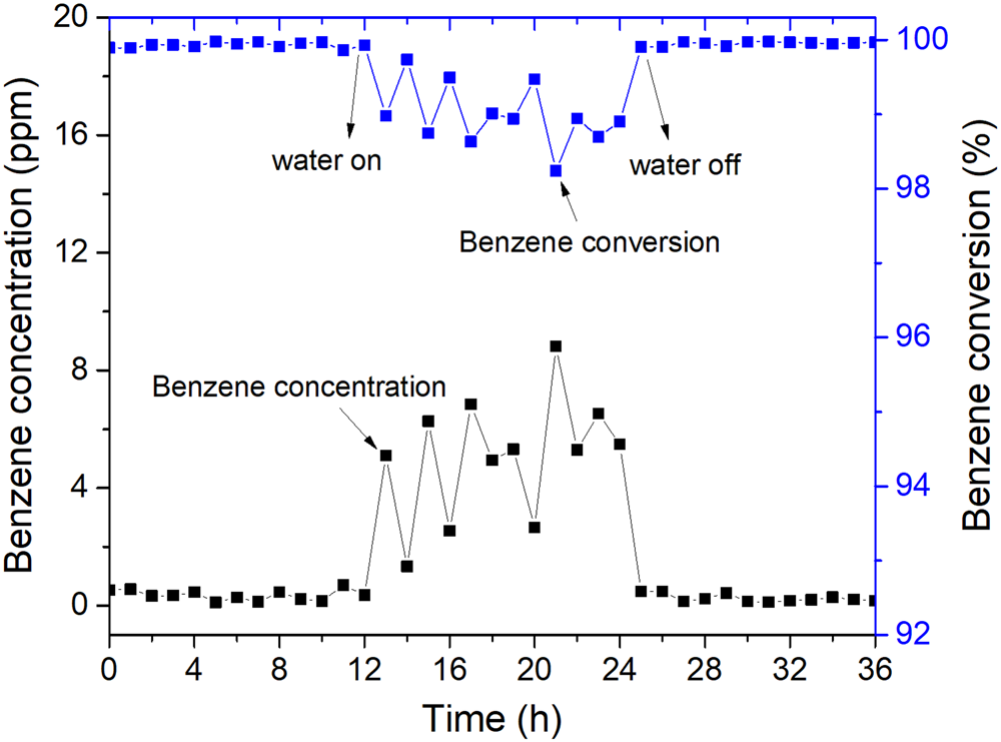
Effect of water vapor (1.5 vol%) on the activity of Pt/SiC@Al_2_O_3_-1 with Pt mass ratio of 0.176% for oxidation of benzene, WHSV = 60 000 mL g^−1^ h^−1^ at 250 °C for 36 h with and without water vapor.

The effect of water vapor (1.5 vol%) on the catalytic performance of the Pt/SiC@Al_2_O_3_ specimen with Pt mass ratio of 0.176% was investigated. As evinced by the benzene conversion curve or by benzene concentration curve shown in Figure 4, at WHSV of 60 000 mL g^−1^ h^−1^ and temperature of 250 °C, there is only ca. 1–2% decrease in benzene conversion after the water vapor is introduced into the reaction system. The inhibition induced by water vapor might be due to the competitive adsorption of water and benzene as well as oxygen molecules^44,45^. Fortunately, the benzene conversion could be restored to 100% after cutting off water vapor.

## Conclusions

In summary, we demonstrated the functionalization of porous SiC ceramics with catalytic capability by loading fine Pt particles on their pre-coated Al_2_O_3_ layers. We found an aqueous salt method-based strategy can render the fine Pt particles to be distributed uniformly on the Al_2_O_3_ layers coated on the SiC substrates. The evaluation for catalytic benzene oxidation shows that at a weight hourly space velocity of 6000 mL g^−1^ h^−1^, the as-extruded Pt/SiC@Al_2_O_3_ rectangular plates with Pt mass loading as low as 0.176% can convert 90% benzene at a temperature of ca. 215 °C. The studies in this work may be promising for the design of bi-functional materials for simultaneously eliminating dusts and harmful VOCs from various hot gases.

## Methods

### General materials

α-SiC powders with average particle size of ca. 167.3 μm from Zhengzhou Xingshi Abrasive Co. Ltd., China, α-Al_2_O_3_ with average particle size of ca. 6.8 μm from Zhengzhou Yufa Abrasive Group Co. Ltd., China, kaolin and methyl cellulose from Sigma-Aldrich, Hydrogen hexachloroplatinate(IV) hydrate (H_2_PtCl_6_·6H_2_O, 37.5% Pt basis), sodium borohydride (NaBH_4_, 98%), tri-sodium citrate dihydrate (C_6_H_5_Na_3_O_7_·2H_2_O, ≥99%), dodecylamine (DDA, 98%), ethanol (99.5%), 2-propanol (97%), polyvinyl alcohol (98%) and toluene (99.5%) from Beijing Chemical Works, were used as received. Glassware and magnetic stirring bars are cleaned with *aqua regia*, followed by copious rinsing with de-ionized water before drying in an oven.

### Preparation of Al_2_O_3_-coated SiC rectangular plates

The rectangular SiC plates were prepared using a protocol reported by Ha *et al*. with modifications^8^. In detail, the SiC powders were mixed with kaolin according to the mass ratio of 92:8, followed by adding 25 wt% of methyl cellulose and 25 wt% of distilled water to form slurries. After aging at room temperature for 48 h, the slurries were extruded using a double screw extruder (SD-150, Zibo, China) into rectangular plates (100 × 10 × 1000 mm) with 10 inner holes (5 × 7 mm). After extrusion and drying for 24 h, the SiC rectangular plates were pre-heated at 400 °C for 1 h to burn-off the organic binder (methyl cellulose), and finally sintered at 1400 °C for 1 h.

For coating SiC rectangular plates with Al_2_O_3_ layers, we firstly mixed the Al_2_O_3_ particles, 2-propanol, distilled water and polyvinyl alcohol with mass ratio of 10:30:57:3, and then ball-milled them for 4 h. Subsequently, the mixtures were dip-coated on the surface of SiC rectangular plates, and the specimens were dried at room temperature for 24 h and heated at 1300 °C for 1 h. The Al_2_O_3_ coating was only conducted on the external surface of the SiC rectangular plates and the thickness of the Al_2_O_3_ layers were controlled by the coating time.

### Loading Pt on the SiC@Al_2_O_3_ rectangular plates

We firstly cut the SiC@Al_2_O_3_ rectangular plates into small pieces with precise weights of 20 g, and then employed three different strategies including aqueous salt method, aqueous particulate method, and organic salt method to load Pt on the SiC@Al_2_O_3_ rectangular plates.

**Figure 5:**
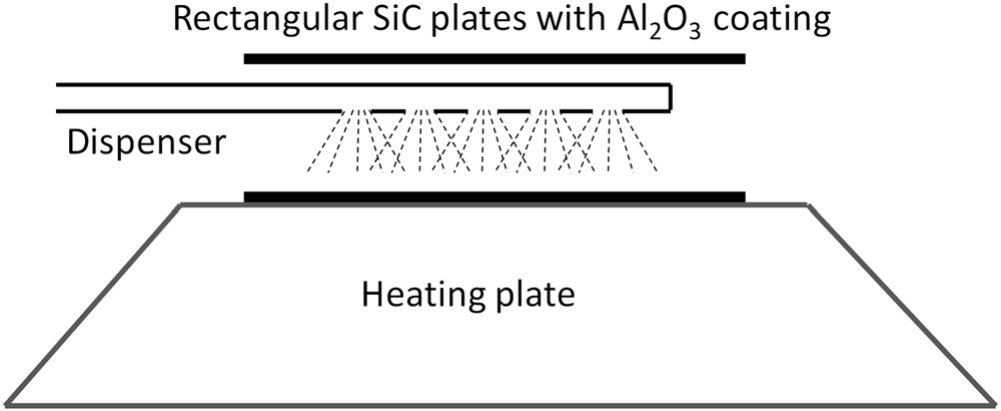
Schematic illustration to show the loading of Pt catalysts on the SiC@Al_2_O_3_ rectangular plates.

Strategy (I)-aqueous salt method: Dissolve 267 mg of H_2_PtCl_6_ (100 mg of Pt) into 10 mL of water, and the solution was evenly dispensed into the inner surface of the SiC@Al_2_O_3_ rectangular plates, which were put on a heating plate, as shown in Figure 5 for the schematic illustration. The water diffused through the wall of SiC@Al_2_O_3_ rectangular plates would be evaporated by the heating plate. After completing the Pt ion loading on both sides, the SiC@Al_2_O_3_ rectangular plates were dried at room temperature, and then heated at 350 °C in a muffle furnace for 2 h under atmosphere.

Strategy (II)-aqueous particle method: Prepare aqueous colloidal Pt solution using NaBH_4_ reduction of H_2_PtCl_6_ in the presence of sodium citrate as stabilizing agent, and load these Pt particles on the SiC@Al_2_O_3_ rectangular plates using the dispenser. In detail, 0.2 mL of a freshly prepared 100 mM aqueous solution of NaBH_4_ was added dropwise to 100 mL of a 2 mM aqueous H_2_PtCl_6_ solution containing 0.8 mmol of sodium citrate to obtain Pt colloid^46^, which was aged for overnight to decompose the residual NaBH_4_, and then deposited on the SiC@Al_2_O_3_ rectangular plates using the setup in Strategy (I). After loading, the SiC@Al_2_O_3_ rectangular plates were also dried and heated at 350 °C in a muffle furnace for 2 h under atmosphere.

Strategy (III)-organic salt method: Dissolve the H_2_PtCl_6_ into water, and transfer the Pt ions into toluene using an ethanol-mediated method^47,48^, followed by loading on SiC@Al_2_O_3_ rectangular plates using the protocol same as Strategy (I). In brief, 100 mL of 2 mM aqueous H_2_PtCl_6_ solution was mixed with 100 mL ethanol containing 4 mL of dodecylamine. After mixing the mixture for 5 minutes, 100 mL of toluene was added, and the stirring was continued for another 3 minutes, followed by collecting the toluene phase after the two phases were completely separated. Finally, the steps in Strategy (I) were followed to fulfill the deposition of Pt on the SiC@Al_2_O_3_ rectangular plates.

The mass ratio of Pt on the SiC@Al_2_O_3_ rectangular plates were tuned by altering the concentration of H_2_PtCl_6_ solution or volume of Pt colloid, and the precise amount of Pt on the porous ceramics were analyzed with inductively coupled plasma-atomic emission spectrometry (ICP-AES, Optima 5300DV, Perkin Elmer, America). The specimens obtained by Strategy (I), (II), and (III) were labeled as Pt/SiC@Al_2_O_3_-1, Pt/SiC@Al_2_O_3_-2, and Pt/SiC@Al_2_O_3_-3, respectively.

### Characterizations of SiC@Al_2_O_3_ and Pt/SiC@Al_2_O_3_ specimens

Scanning electron microscopy (SEM) and transmission electron microscopy (TEM) were carried out on a JEOL-7100F the JEOL JEM-2100F electron microscopes, respectively. Powder X-ray diffraction (XRD) measurements were carried out on a Bruker D8 focus X-ray diffractometer using Cu-K_α_ radiation (λ = 1.5406 Å). X-ray photoelectron spectroscopy (XPS) analysis was conducted on a VG ESCALAB MKII spectrometer.

### Catalytic evaluation of Pt/SiC@Al_2_O_3_ specimens for benzene conversion

We evaluated the catalytic performance of Pt/SiC@Al_2_O_3_ specimens for benzene oxidation using a continuous-fixed-bed quartz microreactor with inner diameter of 4 mm at a weight hourly space velocity (WHSV) of 60 000 mL g^−1^ h^−1^. The SiC@Al_2_O_3_ rectangular plates with Pt loading were broken up into small particles with sizes of 60–80 mesh. The specimens (100 mg) mixed with 200 mg of quartz sand (40–60 mesh) were placed into the quartz reactor with quartz wool packed at both ends of the catalyst bed. The reactant gases composed of 500 ppm gaseous benzene and air (20% O_2_+ balance N_2_) were purged into the reactor at a continuous flow of 100 mL min^−1^. The concentration of benzene was analyzed using a gas chromatograph (Shimadzu GC-2014) equipped with a flame ionization detector (FID). The conversion rate of benzene (*η*_Ben_) was calculated based on the following equation:

1 $${\eta }_{{\rm{Ben}}}=({C}_{\mathrm{Ben},\mathrm{in}}-{C}_{\mathrm{Ben},\mathrm{out}})/{C}_{\mathrm{Ben},\mathrm{in}}\times 100 \% $$

where *C*_Ben,in_ (ppm) and *C*_Ben,out_ (ppm) are the concentrations of benzene in the inlet and outlet gases, respectively.

To investigate the effect of water vapor on the catalytic performance of Pt/SiC@Al_2_O_3_ specimens for benzene oxidation, we passed the air flow containing gaseous benzene (100 mL min^−1^) through a water saturator at a certain temperature so that 1.5 vol% concentration of H_2_O could be introduced into the reaction system. The reactants and products were detected online on a gas chromatograph (Shimadzu GC-2014).

## Electronic supplementary material

Supplementary Information
