# Supplementary material for: Fine platinum nanoparticles supported on a porous ceramic membrane as efficient catalysts for the removal of benzene
Source: Sci Rep. 2017 Nov 29;7:16589. doi: 10.1038/s41598-017-16833-0 (PMC5707434; doi:10.1038/s41598-017-16833-0)
Supplement: Supplementary file 1 — Supplementary Information [file 41598_2017_16833_MOESM1_ESM.doc]

Supplementary Information

**Fine Platinum Nanoparticles Supported on Porous Ceramic Membrane as Efficient Catalysts for the Removal of Benzene**

Hui Liu^1,2^, Chengyin Li^1^, Xiaoyong Ren^1^, Kaiqi Liu^1,*^, and Jun Yang^1,2,3,*^

^1^State Key Laboratory of Multiphase Complex Systems, Institute of Process Engineering, Chinese Academy of Sciences, Beijing 100190 China. Tel: 86-10-8254 4915; Fax: 86-10-8254 4915; Email: [jyang@ipe.ac.cn](mailto:jyang@mail.ipe.ac.cn) (J.Y.); Tel: 86-10-8254 5059; Fax: 86-10-8254 5059; Email: [kqliu@ipe.ac.cn](mailto:jyang@mail.ipe.ac.cn) (K.L.)

^2^Center for Mesoscience, Institute of Process Engineering, Chinese Academy of Sciences, Beijing 100190, China

^3^University of Chinese Academy of Sciences, No. 19A Yuquan Road, Beijing 100049, China

This work was financially supported by National Key R&D Program of China (Grant No.: 2016YFB0601100), National Natural Science Foundation of China (Grant Nos.: 21376247, 21506225, 21573240), and Center for Mesoscience, Institute of Process Engineering, Chinese Academy of Sciences (Grant No.: COM2015A001).

**Figure S1.** The XPS spectrum of Pt in Pt/SiC@Al_2_O_3_-2 specimens. Only Pt 4f_7/2_ was analyzed due to the overlaps between Al 2p and Pt 4f_5/2_ binding energies, and the XPS analysis shows that a small portion of Pt is in the oxidized state.

**Figure S2.** XRD patterns of blank SiC@Al_2_O_3_ rectangular plates (a), Pt/SiC@Al_2_O_3_-1 (b), Pt/SiC@Al_2_O_3_-2 (c), and Pt/SiC@Al_2_O_3_-3 (d). The references with JCPDF Card File of 741302 for SiC, 100173 for Al_2_O_3_, and 870644 for Pt are also displayed.

**Table S1.** Benzene conversion on the blank SiC@Al_2_O_3_ rectangular plates, Pt/SiC@Al_2_O_3_-1, Pt/SiC@Al_2_O_3_-2, and Pt/SiC@Al_2_O_3_-3 specimens. The mass loading of Pt is 0.04%.

| Catalyst | Benzene conversion | | |
| --- | --- | --- | --- |
|  | T_10_ (°C) | T_50_ (°C) | T_90_ (°C) |
| Blank SiC@Al_2_O_3_ | − | − | − |
| Pt/SiC@Al_2_O_3_-1 | 194.2 | 229.5 | 268.2 |
| Pt/SiC@Al_2_O_3_-2 | 216.2 | 251.2 | 306.9 |
| Pt/SiC@Al_2_O_3_-3 | 236.7 | 285.9 | − |

**Table S2.** Performance comparisons of noble metal-based catalysts for benzene oxidation.

| Catalysts | Metal loading (wt%) | Catalyst amount (mg) | Benzene concentration (ppm) | Space velocity (mL g^-1^ h^-1^) | *T_90_* (°C) | Refs. |
| --- | --- | --- | --- | --- | --- | --- |
| Pt/SiC@Al_2_O_3_-1 | 0.04 | 100 | 500 | 60000 | 268.2 | This study |
| Pd/Al_2_O_3_ | 0.81 | 60 | 482 | 30000 | 310.5 | 38 |
| Pd/5%V_2_O_5_/Al_2_O_3_ | 0.89 | 60 | 482 | 30000 | 302 | 38 |
| Pd/10%V_2_O_5_/Al_2_O_3_ | 0.80 | 60 | 482 | 30000 | 269 | 38 |
| Pd/20%V_2_O_5_/Al_2_O_3_ | 0.90 | 60 | 482 | 30000 | 289 | 38 |
| 0.3Pt1Pd/γ-Al_2_O_3_^a^ | 1 | – | 1000 | 15000 | 225 | 39 |
| 0.1Pt2Pd/γ-Al_2_O_3_^a^ | 2 | – | 1000 | 15000 | 238.5 | 39 |
| 0.3Pt2Pd/γ-Al_2_O_3_^a^ | 2 | – | 1000 | 15000 | 222.5 | 39 |
| 1Pt2Pd/γ-Al_2_O_3_^a^ | 2 | – | 1000 | 15000 | 232 | 39 |
| 3Pt2Pd/γ-Al_2_O_3_^a^ | 2 | – | 1000 | 15000 | 233 | 39 |
| 2Pd/γ-Al_2_O_3_ | 2 | – | 1000 | 15000 | 273 | 39 |
| Pd/TiO_2_ | 0.5 | – | 100 | 45000 | 329 | 40 |
| Pd/0.5%V/TiO_2_ | 0.5 | – | 100 | 45000 | 319 | 40 |
| Pd/1.0%V/TiO_2_ | 0.5 | – | 100 | 45000 | 310.5 | 40 |
| Pd/1.5%V/TiO_2_ | 0.5 | – | 100 | 45000 | 295.5 | 40 |
| Pd/3.0%V/TiO_2_ | 0.5 | – | 100 | 45000 | 285 | 40 |
| Pd/Beta | 0.3 | 300 | 1500 | 26000 | 225 | 41 |
| Pd/ZSM-5 | 0.3 | 300 | 1500 | 26000 | 264 | 41 |
| Pd/MCM-48 | 0.3 | 300 | 1500 | 26000 | 332 | 41 |
| Pd/MCM-41 | 0.3 | 300 | 1500 | 26000 | 367 | 41 |
| Pd/SBA-15 | 0.3 | 300 | 1500 | 26000 | 268 | 41 |
| Pd/ZM-5% | 0.97 | 300 | 1500 | 32000 | 209 | 42 |
| Pd/ZM-10% | 0.96 | 300 | 1500 | 32000 | 214 | 42 |
| Pd/ZM-20% | 0.98 | 300 | 1500 | 32000 | 229 | 42 |
| Pd/ZM-30% | 0.97 | 300 | 1500 | 32000 | 237 | 42 |
| Pd/MCM-48 | 0.97 | 300 | 1500 | 32000 | 292 | 42 |
| Pd/ZSM-5 | 0.96 | 300 | 1500 | 32000 | 244 | 42 |

^a^ mPt, m% is the loading of Pt (m = 0.1, 0.3, 1, 3).
